# Antimicrobial Resistance in Africa—How to Relieve the Burden on Family Farmers

**DOI:** 10.3201/eid2710.210076

**Published:** 2021-10

**Authors:** Christian Ducrot, Alexandre Hobeika, Christian Lienhardt, Barbara Wieland, Charlotte Dehays, Alexis Delabouglise, Marion Bordier, Flavie Goutard, Ekta Patel, Muriel Figuié, Marisa Peyre, Arshnee Moodley, François Roger

**Affiliations:** Animals Health Territories Risks Ecosystems Research Unit (ASTRE), Univ Montpellier, Agricultural Research Center for International Development (CIRAD), Institut National de Recherche pour l’Agriculture, l’Alimentation et l’Environnement (INRAE), Montpellier, France (C. Ducrot, C. Dehays, A. Delabouglise, M. Bordier, F. Goutard, M. Peyre, F. Roger);; CIRAD, Marché Organisations Institutions et Stratégies d’Acteurs Research Unit (MOISA), Montpellier (A. Hobeika);; MOISA, Université de Montpellier, Centre International de Hautes Etudes Agronomiques Méditerranéennes–Institut Agronomique Méditerranéen de Montpellier, CIRAD, INRAE, Institut Agro, Montpellier (A. Hobeika, M. Figuié);; Institut de Recherche pour le Développement (IRD), Recherches Translationnelles sur le VIH et les Maladies Infectieuses Research Unit (TransVIHMI), Montpellier (C. Lienhardt);; TransVIHMI, Université de Montpellier, IRD, Institut National de la Santé et de la Recherche Médicale (INSERM), Montpellier (C. Lienhardt);; International Livestock Research Institute, Addis Ababa, Ethiopia (B. Wieland);; CIRAD, ASTRE, Montpellier (C. Dehays, A. Delabouglise, M. Peyre, F. Roger);; Institut Sénégalais de Recherches Agricoles–Laboratoire National de l’Elevage et de Recherches Vétérinaires, Dakar-Hann, Senegal (M. Bordier);; Kasetsart University Faculty of Veterinary Medicine, Bangkok, Thailand (F. Goutard);; International Livestock Research Institute, Nairobi, Kenya (E. Patel, A. Moodley);; Universidade Eduardo Mondlane Faculdade de Letras e Ciências Sociais, Maputo, Mozambique (M. Figuié);; University of Copenhagen Department of Veterinary and Animal Sciences, Frederiksberg, Denmark (A. Moodley)

**Keywords:** antimicrobial resistance, antimicrobials, Africa, antibiotic resistance, family farmers, farms animals

## Abstract

Although currently available data indicate that Africa has the lowest usage of antimicrobials in animals in the world (adjusted by animal biomass), data show a high prevalence of antimicrobial resistance in foodborne pathogens isolated from animals and animal products. Apart from the lack of solid data on antimicrobial use in many countries in Africa, different hypotheses could explain this situation. Qualitative interviews of farmers show a lack of knowledge and uninformed use of antimicrobials. Considering the development of animal farming to meet an increasing demand for proteins, this deficiency represents a serious public health issue. We advocate for policies that consider the specific challenges faced by family farmers in Africa, to simultaneously improve access to veterinary drugs while strengthening the regulation of their use. We propose a global approach targeting the agri-food system, offering innovative social and technical interventions on antimicrobial usage, adapted to family farmers.

According to international official data, Africa seems to have the lowest usage of antimicrobials in animals in the world, adjusted by animal biomass (*1*), which would tend to indicate that antimicrobial resistance is not a question there. However, an extensive review by Van Boeckel et al. reports that a high prevalence of antimicrobial resistance (AMR) has been observed in foodborne pathogens isolated from animals and animal products in Africa (*2*). This article explores this apparent paradox, which, in fact, points to the quality of available data and the practices about antimicrobial use (AMU).

## Antimicrobial Resistance in Animals in Africa

The literature review by Van Boeckel et al. shows that, in poultry, resistance rates to tetracycline reach 70% for *Escherichia coli* and 80% for *Campylobacter* in Africa, above the rates observed in Asia or the Americas (*2*). Resistance to sulfamethoxazole/trimethoprim and ampicillin is ≈60% for *E. coli*, again above the rates in other continents. The same trend was observed in studies on pigs, ruminants, and even wildlife (*3*), and resistance rates have been increasing over the years (*2*). Fortunately, lower rates of resistance are observed for antimicrobials considered critical to human medicine, such as cephalosporins, although these rates are frequently above those observed globally. However, we should note that scientific data on AMR in animals and animal products was lacking in 10 of the 54 countries in Africa, and 11 of those that provided data experienced challenges because of a lack of experience in collecting data. Although solid data on AMR in different countries in Africa is lacking, the collection of data is improving rapidly.

The absence of national AMU surveillance programs in countries in Africa raises questions about the reliability of reported data, especially because some countries do not provide data to the World Organisation for Animal Health (OIE). Furthermore, data in certain countries probably do not fully capture actual AMU because of illegal sales and undocumented imports of antimicrobials (*1*). However, some studies indicate declining trends in AMU. Thus, Klein et al. mention that, during 2000–2015, antimicrobial consumption in low- and middle-income countries was approaching levels observed in high-income countries (*4*). However, regardless of whether antimicrobial consumption is underestimated in Africa or not, AMR in animal food products is at a high level. Hypotheses that could explain this situation need to be formulated and tested.

To help clarify smallholder farmers’ motivations to use antimicrobials and the barriers they face, qualitative interviews of farmers, studies using participatory methods, and structured studies such as knowledge, attitudes, and practices analyses have been published in recent years. The results of these small-scale studies highlight low levels of AMU awareness and knowledge, AMU that appears to be far from informed or prudent, and AMR risk perceptions that are poor (*5*–*7*; Z.I. Kimera et al., unpub. data, https://doi.org/10.21203/rs.3.rs-33311/v1). Most farmers report using antimicrobials on individual animals to treat a disease but also on their entire flocks and herds to prevent disease and to promote growth (*8*). The prescribed dosage is often not respected (*9*–*11*). Farmers typically use antimicrobials without advice from an animal-health worker (*12*) and can easily purchase drugs without a prescription (*11*,*13*). Most farmers are not aware of the withdrawal period rule (*11*), which stipulates a period between drug administration and the slaughter or consumption of food from the treated animal; the period is defined for each drug to ensure that the food does not contain residue of that drug. Among farmers who report knowing about withdrawal periods, many do not always respect them (*12*,*14*), which sheds light on why various studies have found antimicrobial residues in animal food products (*15*,*16*). In addition, counterfeit and subquality drugs appear to be widespread in low-income countries; ≈60% of antimicrobials in Africa are of poor quality (*17*), which can select for antimicrobial-resistant pathogens. Together, those factors might be contributing to the high level of AMR rates in animals and animal food products in Africa. Additional factors might exist, such as a high burden of infections coupled with poor animal husbandry practices spreading the resistance genes (*18*), addition of antimicrobials to preserve animal products (*19*), weak enforcement of regulations (*20*), and use of antimicrobials in plant production (a practice that is poorly documented and possibly used on a great variety of crops) (*21*). Although the transmission routes of AMR from animals to humans and vice versa are insufficiently investigated in Africa, the situation is of public health concern because of the probability that resistance is being transmitted to farmers and consumers through animals and animal food products and through consumption of antimicrobial residues in animal products. This zoonotic contribution of AMR adds to the high level of AMR to commonly prescribed antimicrobial drugs that have already been observed in humans (*22*). Furthermore, the contribution of environmental contamination through the use of antimicrobials in animal husbandry is poorly documented in Africa, but it could play an important role in the acquisition of resistance in humans. In fact, 90% of antimicrobials administered orally are excreted in urine or manure in a very slightly degraded form and end up in water used for animal and vegetable production (*23*).

Animal farming, especially poultry, is expanding in Africa; meat production has increased by 64% since 2000, in response to an increasing demand for protein by the growing population (*2*,*24*). A major need exists to rationalize the use of antimicrobials for animals in Africa, keeping in mind 3 important aspects. First, and similar to what is observed in other continents, a large proportion of antimicrobial drugs are currently used without a relevant indication, so there is room for a substantial decrease of AMU without negatively affecting the productivity of the livestock industry. Second, antimicrobials remain essential in the treatment of livestock diseases for which no affordable alternative treatment exists; this point is particularly important because livestock provide a range of essential services to society, including income and savings for the rural poor, access to protein, transportation, and manure for fertilizer (*25*–*27*). Third, farming systems in Africa represent a wide continuum ranging from family farms (smallholders) to commercial farms, which have very different levels of access to antimicrobials and support services. Although commercial farmers can adapt to evolving standards, adaptation is more difficult for the smallholder farmers (*28*). Therefore, policies must differentiate between commercial and family farms and be designed to minimize adverse health and socioeconomic effects on family farmers’ standards of living, especially in the most vulnerable areas.

Antimicrobials have the double advantage of being relatively inexpensive and of having highly reliable effects on animal health. Substitutes for antimicrobials were evaluated in the context of large-scale commercial farming systems in high-income countries. These substitutes include investments in long-term prevention plans with vaccination and farm biosecurity enhancement aimed at reducing the probability of bacterial infections (*29*). However, implementing these measures involves training, specialization, and investment that are mostly incompatible with the economic rationale of small-scale farmers, who represent the bulk of the livestock production in Africa (*30*). In the context of most sub-Saharan Africa countries, where the availability of capital, credit, and government support is limited, livestock farming is favored precisely because it requires limited investment, and farmers often tend to diversify their activities to mitigate economic risks rather than specialize in a single product (*25*,*31*,*32*). Access to antimicrobials remains necessary to treat sick animals and thus maintain overall health. The annual cost of infectious animal diseases in Africa is estimated to be US $9.35 billion, and losses caused by lack of treatment far outweigh losses caused by AMR (*33*). Subsequently, in the absence of generalized improvement of access to veterinary services, drastically reducing the use of antimicrobials, which is necessary in the fight against AMR, will probably lead to an upsurge of diseases and a drop in production. This reduction will have an economic impact on small farmers and will reduce access to cheap meat for the most vulnerable groups, resulting in serious adverse effects on their nutrition (*34*). Therefore, the national regulations put in place to curb the emergence of resistance need to be reinforced, take into account the needs of family farmers and low-income populations, and address properly the ethical issues of social values and equal access (*34*).

## The Way Forward

We advocate for policies that consider the specific challenges faced by family farms and commercial farms. On the one hand, the use of antimicrobials by commercial farms needs to be regulated. On the other hand, family farmers face severe difficulties in accessing veterinary drugs and professional veterinary advice (G. Jaime et al., unpub. data, https://doi.org/10.31730/osf.io/8vcj2), which hampers their capacity to change their animal health practices, adopt innovations, and cope with more demanding regulations and production standards. Policies designed to control AMR must meet the dual challenge of simultaneously improving access to veterinary drugs while strengthening the regulation of their use. Moreover, although antimicrobial use in farming is driven by the characteristics of individual farmers and farming systems, it also depends on the functioning of the antimicrobial value chain, existing regulations, and access to veterinary extension services. Consequently, policies should not stigmatize farmers and load them with a burden of measures against AMR but should modify the organization of international and national veterinary drug markets. We are arguing in favor of strong public policies that reinforce access to veterinary services for family farmers and a prudent use of antimicrobials. Efforts must be made to fill gaps in knowledge on the socioeconomic context that prompt farmers to misuse antimicrobials and increase the health risks for their families and consumers. We propose combining several complementary approaches at the farm and market value chain levels.

First, we urge using actions adapted to the complexity of agri-food systems (*35*), which entails assessing agricultural systems, identifying and understanding livestock farming networks and processing methods (with particular emphasis on practices linked to antimicrobials), developing programs of action aimed at rationalizing antimicrobial use on the basis of a participatory approach joining researchers with all the stakeholders involved, and targeting priority actions according to sectors and geographic areas.

Second, we advocate better regulation of farming practices that improves farmers’ access to training (with special attention to sex) and advice (or support services) provided by trained veterinarians or paravets. Regulation should also be aimed at increasing biosecurity on farms (*36*), including better control of the introduction of animals, inputs, and drugs, notably the use of growth promoters; more effective management of contacts between animal species; closer monitoring of conditions in farm buildings; and vaccination against common infectious diseases to reduce the need for antimicrobials to treat preventable infections. We also recommend education and training programs for veterinarians and paraveterinary and community-based animal health workers, in close collaboration with official authorities and nongovernment organizations such as Agronomes et Vétérinaires Sans Frontières, with the aim of promoting a reasoned use of antimicrobials and expanding the offer of veterinary services.

We recommend better structuring, regulation, and monitoring of the flows of veterinary drugs through improving farmers’ access to medicines, especially in rural areas; improving information about drug quality from importation to local distribution; and overall monitoring of the flows of medicines. We also call for regulating and controlling the sale of illegal drugs, with the support of governing bodies such as OIE, as well as establishing appropriate, integrated, and multisectoral monitoring of AMU trends and resistance to antimicrobials in animals, food products, and the environment (*37*).

In practice, a major difficulty in fair public health reforms, which involve and affect a wide range of actors, is generally a lack of information about the political and social context in which they take place. Mapping antimicrobial supply chains could help overcome this problem by identifying target groups, their ability to block or support reform, and their ability to ally with and influence the political process (through power, leadership, or both) and to propose strategies to promote supportive actions and reduce counter actions (*38*). After this mapping, innovative social and technical interventions on AMU, adapted to the socioeconomic level of smallholders, could be co-constructed by using, for example, participatory modeling (*39*). The main idea of participatory modeling is to make explicit biological processes and the strategies and social relationships of the actors involved so that the actors themselves can deal with their own problems and identify mutually acceptable solutions that can lead to collective action plans. The efficacy of these co-constructed interventions could be assessed with randomized cluster trials to determine which measures have an impact, the magnitude of that impact, which strategies are most cost-effective, and which ones can be adopted quickly. Developing strong public–private partnerships within this process involving farmers, public and private veterinarians, public authorities, drug companies, and agro-industries, would be key to ensure the sustainability of the actions. Private organizations included in the partnership might support the process in the long run and ensure proper implementation of best practices for AMU as well as surveillance and control measures (e.g., vaccination and biosecurity) (*40*).

## Conclusion

Reducing antimicrobial consumption and misuse is critical for reducing the threat of AMR in Africa. To that end, considering the complexity of social-ecologic systems, analyzing AMU drivers, monitoring AMR and AMU, and involving stakeholders in participatory approaches (in a One Health framework) will be key to ensuring the efficiency of action plans and regulations and to safeguarding family farming communities. Such reduction efforts must be linked with improved access to nonmedicated animal feed and better access and use of antimicrobials that are needed to treat sick animals. Similar to programs used in human health, antimicrobial stewardship programs geared around family farmers and involving all stakeholders should be developed and put in place in Africa (*41*), taking into account the specificities of the situation in each country.
